# Excision of sympathetic ganglia and the rami communicantes with histological confirmation offers better early and late outcomes in Video assisted thoracoscopic sympathectomy

**DOI:** 10.1186/1749-8090-3-50

**Published:** 2008-08-13

**Authors:** Sridhar Rathinam, Prakash Nanjaiah, Sivakumar Sivalingam, Pala B Rajesh

**Affiliations:** 1 Regional Department of Thoracic Surgery, Birmingham Heartlands Hospital, Bordesley Green East, Birmingham, B9 5SS, UK

## Abstract

**Background:**

Video-Assisted Thoracoscopic Sympathectomy (VATS) is an established minimally invasive procedure for thoracic sympathetic blockade in patients with hyperhidrosis, facial flushing and intractable angina. Various techniques using clips, diathermy and excision are used to perform sympathectomy. We present our technique of excision of the sympathetic chain with histological proof and the analysis of the early and late outcomes.

**Methods:**

We evaluated 200 procedures in 100 consecutive patients, who underwent Video Assisted Thoracoscopic Sympathectomy by a single surgeon in our centre between September 1996 to March 2007. All patients had maximum medical therapy prior to surgery and were divided into 3 groups based on indications, Group 1(hyperhidrosis: 48 patients), Group 2 (facial flushing: 26 patients) and Group 3(intractable angina: 26 patients). The demography and severity of symptoms for each group were analysed. The endpoints were success rate, 30 day mortality, complications and patient's satisfaction.

**Results:**

99 patients had bilateral VATS sympathectomy and 1 had unilateral sympathectomy. The conversion rate to open was 1(1%). All patients had successful removal of ganglia proven histologically with no perioperative mortality in our series. The complications included pneumothorax (5%), acute coronary syndrome (2%), transient Horner's syndrome (1%), transient paraesthesia (1%), wound infection (4%), compensatory hyperhidrosis (18%), residual flushing (3%) and wound pain (5%). There were five late deaths in the intractable angina group at a mean follow up of 36.7 months. Overall success rates of abolishing the symptoms were 96.3%, 87.5% and 95.2% for Group 1, 2 and 3 respectively.

**Conclusion:**

Excision of the sympathetic chain with histological confirmation during VATS sympathectomy is a safe and effective method in treating hyperhidrosis, facial flushing and intractable angina with good long term results and satisfaction.

## Background

Thoracoscopic sympathectomy has established itself as a procedure for a thoracic surgeon since it was first reported by Hughues in 1942[1] and popularized by Kux [2]. This was first introduced in 1996 at our regional thoracic surgical unit for the treatment of 3 conditions namely hyperhidrosis, facial flushing and intractable angina. We report our experience of the first hundred patients who had resection of T2 – T4 sympathetic ganglia along with the rami communicantes performed by a single surgeon and their early and long term outcomes.

## Methods

**Figure 1:**
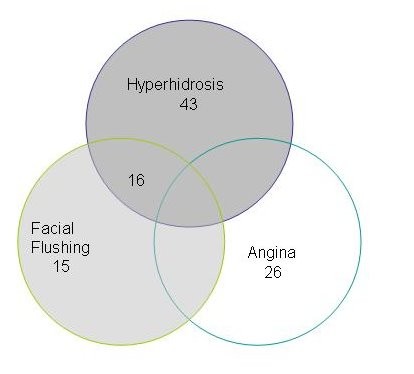
The Distribution of cases in the various groups.

A retrospective review of prospectively collected data was performed on Video Assisted Thoracoscopic Sympathectomies with histological proof of excision to review early and late outcomes. 100 patients underwent 200 VATS sympathectomy by a single surgeon in a tertiary thoracic centre between September 1996 and March 2007 There were 47 males(47%) with a mean age of 32 years (range 18–80) All patients had maximum medical therapy prior to surgery. They were classified into 3 groups based on indications, Group 1: hyperhidrosis, Group 2: facial flushing and Group 3: intractable angina. The distribution of the cases has been illustrated in figure 1. The study was approved by the department and discussed with the ethics committee which advised ethics approval was not required as this was an outcome audit. Diagnosis was made in all patients by history and examination. All patients who underwent the procedure had subjective symptoms affecting quality of life. The patients in the hyperhidrosis group had an unsuccessful trial of medical therapy. Their symptoms were scaled on the basis of symptoms and impact on quality of life (Grade 1: minimal impact on their QOL, Grade 2: caused significant impact on quality of life and Grade 3: severe impact on quality of life). Surgery was only offered to patients with grade 2 and grade 3 symptoms. In the angina group, patients were referred with angina refractory to maximal anti-anginal therapy and deemed unsuitable anatomically for coronary revascularisation. Initial assessment included a detailed history with regards to angina symptoms, degree of disability and effects on quality of life. Patients had a pre-study exercise tolerance test to assess exercise capacity and confirm objective evidence of ischaemia. MUGA scans or transthoracic echocardiogram was performed to determine the left ventricular function. Each patient's angiogram was reviewed by an independent cardiologist and cardiothoracic surgeon and confirmed to be unsuitable for coronary artery bypass grafting or percutaneous cardiological intervention.

**Table 1:** Patient Satisfaction Outcomes

Group	No change	Better	Very satisfied	% improved
Hyperhidrosis (48)	2	9	37	96%
Facial flushing (26)	4	9	13	85%
Intractable angina (26)	2	6	18	92%

### Procedure

Bilateral VATS sympathectomy was performed under general anaesthesia with single lung ventilation starting with the left side followed by right. We found this approach helpful in avoiding arrhythmias in patients with intractable angina. All the patients had electrocardiogram, pulse oximetry and blood pressure monitored during the procedure. In addition the patients undergoing it for angina had invasive arterial monitoring as well (in our initial practice we routinely placed a pulmonary artery floatation catheter in all patients undergoing sympathectomy for angina). A slight degree of cranial elevation and the lateral thoracotomy position helps the lungs to drop away from the operating site exposing the sympathetic chain. The first port was placed in the 5th intercostal space below and anterior to inferior angle of scapula. A 10 mm zero degree telescope was passed through this port. Two further ports were placed for instrumentation at the level of the 3rd and 4th intercostal spaces in the anterior axillary line. The parietal pleura was then incised to expose the sympathetic chain. An extensive thoracic sympathectomy was performed using electrocautery and excision of thoracic sympathetic chain from 2nd to 4th ganglia along with associated rami communicantes. In patients with hyperhidrosis and facial flushing the parietal pleura was cleared for 2 cm lateral to the sympathetic chain in the 2nd intercostal space. This was performed to identify the accessory nerve of Kuntz which was then ablated. The excised ganglia are confirmed histologically in each case. A redivac drain was placed in the apex through the anterior port. The wound was closed in layers with 2'0' vicryl on a 'J-shaped' needle for the intercostal muscle layer, 2'0' vicryl for the subcutaneous tissue and 3'0' monocryl for the skin. Local anaesthetic was infiltrated to the port sites. The patient was then repositioned and the procedure was repeated on the contra lateral side.

All the patients were extubated on table and were nursed in the thoracic surgical ward except the angina patients who were nursed in the Coronary Care Unit. Patient controlled morphine analgesia was provided for pain relief. The redivac drains were removed the day after the operation if the lungs were satisfactorily expanded. Follow-up was made by outpatient visit, medical notes review, and a telephone interview of patients who were discharged from our care. Patients were asked to rate their operative outcome of the procedure as 1 for no change, 2 for satisfactory and 3 to denote a significant improvement and its impact on their quality of life.

## Results

In the time frame study 100 patients underwent 200 VATS sympathectomies. Ninety nine patients had bilateral procedures (one unilateral re operation after 4 years) and one patient had unilateral procedure. There were no post-operative deaths. There was conversion to thoracotomy in the angina group in one patient who had a previous decortication. The median post operative length of stay was 2.4 days for the facial flushing and hyperhidrosis groups and 5.1 days for the angina group. Early complications included acute coronary syndrome, pneumothorax, seroma, transient Horner's syndrome, transient paraesthesia and wound infection as detailed in Table 2. Compensatory hyperhidrosis occurred in 18 patients and was not severe enough to affect their quality of life.

**Table 2:** Early complications

Complication	Number of patients	%
Acute coronary syndrome	2	2%
Pneumothorax	5	5%
Transient Horner's Syndrome	1	1%
Transient Parasthesia	1	1%
Compensatory hyperhidrosis	18	18%
Wound infection	4	4%
Seroma	1	1%

**Figure 2:**
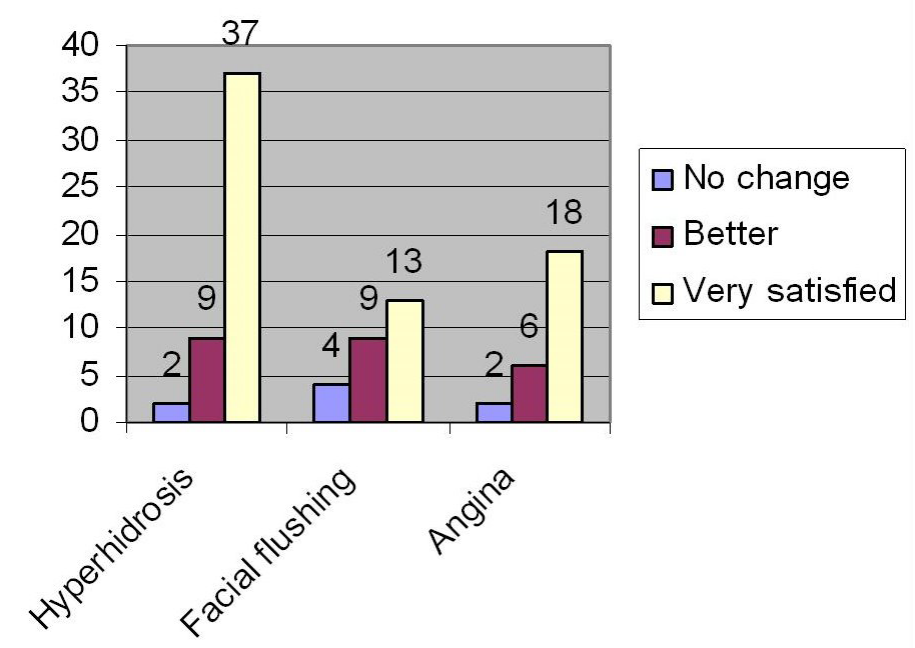
The patient satisfaction outcomes.

The patients were subjectively assessed for their symptoms in the out patients clinic at 4 weeks, 3 months and 6 months and the care was transferred to the referring clinician. Late outcomes were obtained through telephone-conducted patient interviews. All patients were graded for their improvement in the symptoms as 1 for no change, 2 for better and 3 for satisfied. In the hyperhidrosis group 46 patients (96%) were satisfied with the procedure. In the facial flushing group 22(85%) were satisfied with the procedure (Figure 2).

The angina group were assessed by their pre and post procedure Canadian Cardiovascular score for angina, subjective angina score out of 10 and frequency of anginal episodes. Of the twenty five patients who underwent the procedure for relief of refractory angina only one patient (4%) did not feel any difference all the rest had an immediate relief in their symptoms. One patient had atrial fibrillation and two had a post operative myocardial infarction in the early post operative phase.

### Follow up

Full follow-up was available for a mean 67.8 months. In the angina group there were 5 deaths at a mean of 28.75 +/- 13.7 months after procedure. Of these, 2 deaths were cardiac related deaths, one patient died of lung cancer, one patient due to perforated bowel and one died of terminal colonic cancer. The patients who died had symptomatic relief and were satisfied with the operation. On late follow-up 1 patient continued to have anginal symptoms and there was recurrence of angina in two patients at a follow up of 3 and 36 months. Of these patients with recurrence one patient the angina symptoms were present only on the right side and one patient had a dorsal spinal cord stimulator fitted which offered him relief.

In the hyperhidrosis group one patient was noted to have unilateral persistence of sweating and he underwent a re do right sympathectomy after four years which abolished his symptoms. In the original operation the T 2 sympathectomy was not performed on the right due to the presence of large veins this was remedied in the second operation. 4 (15%) patients of facial flushing group had residual facial flushing. 18 patients (18%) had compensatory hyperhidrosis but none found this complication to adversely affect their quality of life. Five patients had post thoracoscopic pain of which one had chronic pain needing referral to the pain clinic. The patient satisfaction results are tabulated in table 1/figure 2.

## Discussion

VATS sympathectomy is an established therapeutic option for hyperhidrosis [3], facial flushing [4], Raynaud's disease [5] and ischaemic heart disease [6].

Primary or essential Hyperhidrosis is a functionally, professionally, and socially disabling condition. It is a pathological condition characterized by excessive secretion of the eccrine glands resulting in overpespiration disproportionate to the requirements of thermoregulation and dissipation of body heat [7]. The current medical treatment modalities for hyperhidrosis include topical application of aluminium salts, tap water iontophoresis, botulinum toxin injections, behavioural and psychotherapy with limited success [8]. The pathophysiology of facial flushing is uncertain, but it is thought to involve vasomotor and sudomotor imbalances [4].

In patients unsuitable for surgical intervention but with intractable angina therapeutic options included long-term intermittent urokinase, spinal cord stimulation and Trans Myocardial Revascularisation. Wettervik showed that VATS sympathectomy had encouraging early results in this group of patient [9]. Benefit is thought to be related to pain anaesthesia of the upper thoracic sympathetic afferent and efferent fibres[10] and vasodilatation following coronary blockade of alpha adreno receptor mediated sympathetic vasoconstriction [11].

Several groups have adopted a single or two port technique for VATS sympathectomy [7,12,13]. In our series we performed the procedure with the 3-port technique, since it allows accurate, safe and reproducible dissection of the thoracic sympathetic chain. We use conventional Thoracoscopic instruments (i.e. no articulating instruments) and training residents is much easier with the three port technique.

Excision of the appropriate sympathetic ganglia is essential for effective post operative outcomes. Excision of the lower third of the stellate ganglia increases the risk of Horner's syndrome [14]. Removal of ganglia between T2 – T4 is optimal for sympathetic denervation of the upper limb [15]. In the treatment of intractable angina we have shown that T2 – T4 sympathectomy was sufficient to alleviate symptoms without risking undue hypotension and Horner's syndrome associated with more extensive excision. Excision of the second to fourth sympathetic ganglia as opposed to clipping [16] or electro coagulation [17] may reduce the incidence of recurrence. In patients with hyperhidrosis and facial flushing, the nerve of Kuntz was excised to reduce recurrence[18,19].

The cause for late recurrence after a successful sympathectomy involves sensitization produced by section of postganglionic fibres. In our study the recurrence of hyperhidrosis was 2% which is comparable to that reported in the literature 6.5% reported in literature [20]. The most common complication of thoracic sympathectomy is transient compensatory sweating probably due to thermoregulatory imbalance. This occurred in 18% of the patients in our study which is consistent with the reported incidence of 20% to 86% [21-23]. We feel the major adverse complications in our series are less due to the three port technique and better visualisation. We did not have any vascular injuries, reexplorations for bleeding, permanent Horner's syndrome or chylothorax which are reported in the literature [22].

We have demonstrated that patients with intractable angina treated with VATS sympathectomy, had a decrease in angina score and frequency of angina as well as an improvement in exercise tolerance [6]. One of the concerns with this procedure was that the denervation of the heart might result in silent ischaemia and mortality, which has not been the case. The other concern was if denervation leads to symptom relief why this was not a complete abolition of symptoms. We feel the benefits are individualised because the sympathetic innervation is collateralised from the aortic plexus which derives its fibres from the cervical plexus. In this current study, we have studied the long term outcomes of the same and noted persistent benefit in symptom alleviation. VATS sympathectomy is a durable palliation for symptoms of angina in a group with few other reliable therapeutic options.

It is our policy to send the excised sympathetic chain for histological confirmation as a quality control measure. We feel this is a good practice in the current medico legal climate so that the surgeon can demonstrate the chain was excised.

## Conclusion

Video assisted thoracoscopic sympathectomy with histological proof of excision is a safe and effective procedure attended by low complications. It is an effective treatment for hyperhidrosis and facial flushing with good long term benefits and patient satisfaction. It offers lasting relief from angina and improves quality of life in patients with intractable angina without options of conventional revascularisation. Histological confirmation of the excised sympathetic chain is valuable for medico-legal documentation.

## Abbreviations

VATS: Video assisted Thoracoscopic surgery; QOL: Quality of Life

## Competing interests

The authors declare that they have no competing interests.

## Authors' contributions

SR was involved with study design, performed the data analysis and authored the manuscript, PN was involved in data collection and follow up, SS designed the study, collected the data and performed data analysis and PBR devised the study, performed all the operations and co authored the manuscript. All authors have read and approved the manuscript.
